# A case study of long-term disease burden in a rural community near an open burn facility

**DOI:** 10.3389/ebm.2025.10710

**Published:** 2025-09-18

**Authors:** Arundhati Bakshi, Liana Baconguis, Md Abdullah Al-Mamun, Qingzhao Yu, Jennifer Richmond-Bryant, Stephania A. Cormier

**Affiliations:** ^1^ Department of Biological Sciences, Louisiana State University, Baton Rouge, LA, United States; ^2^ Department of Biostatistics and Data Science, Louisiana State University Health Sciences Center, New Orleans, LA, United States; ^3^ Department of Forestry and Environmental Resources, North Carolina State University, Raleigh, NC, United States; ^4^ Pennington Biomedical Research Center, Baton Rouge, LA, United States

**Keywords:** open burning, hazardous waste, rural communities, environmental health, health impact

## Abstract

Open burning and open detonation (OB/OD) of explosive and hazardous wastes creates various toxic waste products, including particulate matter, that is released into the atmosphere and capable of generating significant health impacts upon exposure. The last commercially run OB/OD thermal treatment facility in operation in the United States is located near the rural community of Colfax in central Louisiana. To evaluate the community’s concerns about the potential health impacts from air pollution due to the facility’s regular open burning of explosive and hazardous wastes, we examined the disease burden in Colfax compared to the surrounding parish and state. In a cross-sectional study, we analyzed hospitalizations and mortality (2000–2018) where a primary or secondary disease code was associated with cardiovascular, respiratory, thyroid and skin disease. After adjusting for age, sex and race, morbidity and mortality due to cardiovascular and respiratory diseases were significantly higher in Colfax compared to the surrounding areas. In addition, comparing age-adjusted rates across geographies, stratified by race and sex, revealed place-based differences within sub-populations. The higher estimated prevalence of disease conditions is consistent with long-term particulate matter exposure and suggests a need for comprehensive exposure studies within the community. Our data further stress the need for enhanced epidemiological studies and tailored statistical methods to address exposures and environmental health impacts in rural populations, with fewer than 2,500 individuals, like Colfax.

## Impact Statement

This study investigates the health impacts associated with the last remaining commercial open burning/open detonation (OB/OD) facility in the United States, located in the rural town of Colfax, Louisiana. The facility's ongoing open burning of explosive and hazardous waste has raised significant concern among residents about long-term exposure to particulate matter and other toxic byproducts.We present a cross-sectional analysis of hospitalization and mortality data (2000–2018) for cardiovascular, respiratory, thyroid, and skin diseases in Colfax, comparing these rates to the surrounding parish and the state. This study was driven by residents citing various respiratory, cardiovascular, dermal, and thyroid disorders, in addition to cancers. Our findings demonstrate significantly elevated rates of cardiovascular and respiratory diseases, consistent with long-term exposure to airborne pollutants from OB/OD activities. Importantly, we also highlight disparities within subpopulations when stratifying by race and sex, underscoring the necessity of context-specific public health interventions. This work provides a rare and timely evaluation of pollutant exposure and health outcomes in a rural, underserved community. Moreover, it emphasizes the urgent need for robust epidemiological methods tailored to small populations that often fall outside the scope of traditional environmental health surveillance.We believe our study offers critical insights into the environmental justice implications of hazardous waste disposal practices and the value of integrating community concerns into scientific investigation.

## Introduction

Thermal treatment of explosives and other hazardous wastes by open burning and open detonation (OB/OD) results in incomplete combustion of the wastes and the dispersal of residual hazardous constituents into the air. Thus, people may be exposed to hazardous constituents by inhalation of contaminated air, ingestion of contaminated water and food, and/or dermal contact and absorption. While the residual constituents released depend on the compounds processed by the OB/OD facility, studies of military burn pits with explosive waste streams have identified emissions of respirable particulate matter (PM) along with associated environmentally persistent free radicals, which act as precursors to dioxins and furans, polycyclic aromatic hydrocarbons, metals, and volatile organic compounds [1–6]. In addition to irritation of the respiratory tract, eyes, and skin, human exposure to these substances have been associated with increased cancer rates and respiratory, cardiovascular, endocrine, and neurologic diseases [7–10].

Colfax is a rural town in the state of Louisiana and is the seat of Grant Parish (the state’s equivalent to counties). According to the U.S. Census Bureau’s American Community Survey (2022), it has a population of about 1600 people and a higher percentage of Black residents (66%) than Grant Parish (15%) and Louisiana (32%). A larger percentage of the Colfax population lives below poverty level (38%) compared with Grant Parish (15%) and Louisiana (19%) [11] (Table 1).

**TABLE 1:** Comparison of American Community Survey 5-year estimates (2022) for Louisiana, Grant Parish, Colfax town, ZIP code 71417 (which includes Colfax and is defined as Colfax for this study), and other ZIP codes in Grant Parish.

Characteristics	Louisiana	Grant Parish	ZIP code 71417 (Colfax)^a^	All other Grant Parish ZIP codes^c^
Total population	4,640,546	22,185	4,943	18,434
% Age <18 years	23%	21%	21%	20%
% Age ≥65 years	16%	15%	18%	15%
% Female	51%	43%	44%	43%
% All non-White races (% Black or African-American alone)	41% (32%)	21% (15%)	32% (31%)	17% (11%)
% Hispanic or Latino	6%	5%	1%	6%
% Below Poverty Level	19%	15%	21%	12%
% Without health insurance^b^	8%	10%	7%	11%
% Covered by Medicaid alone^b^	23%	29%	31%	27%

^a^ Data pertain to ZIP Code Tabulation Areas (ZCTAs), which are the closest geographic approximation of ZIP codes. As ZCTAs can cross state and parish boundaries, the sum of the population by ZCTA may not equal to the census population data for the parish.

^b^ Data pertain to civilian, non-institutionalized population only.

^c^ Includes ZIP codes 71404, 71407, 71423, 71432, 71454, 71467.

**FIGURE 1:**
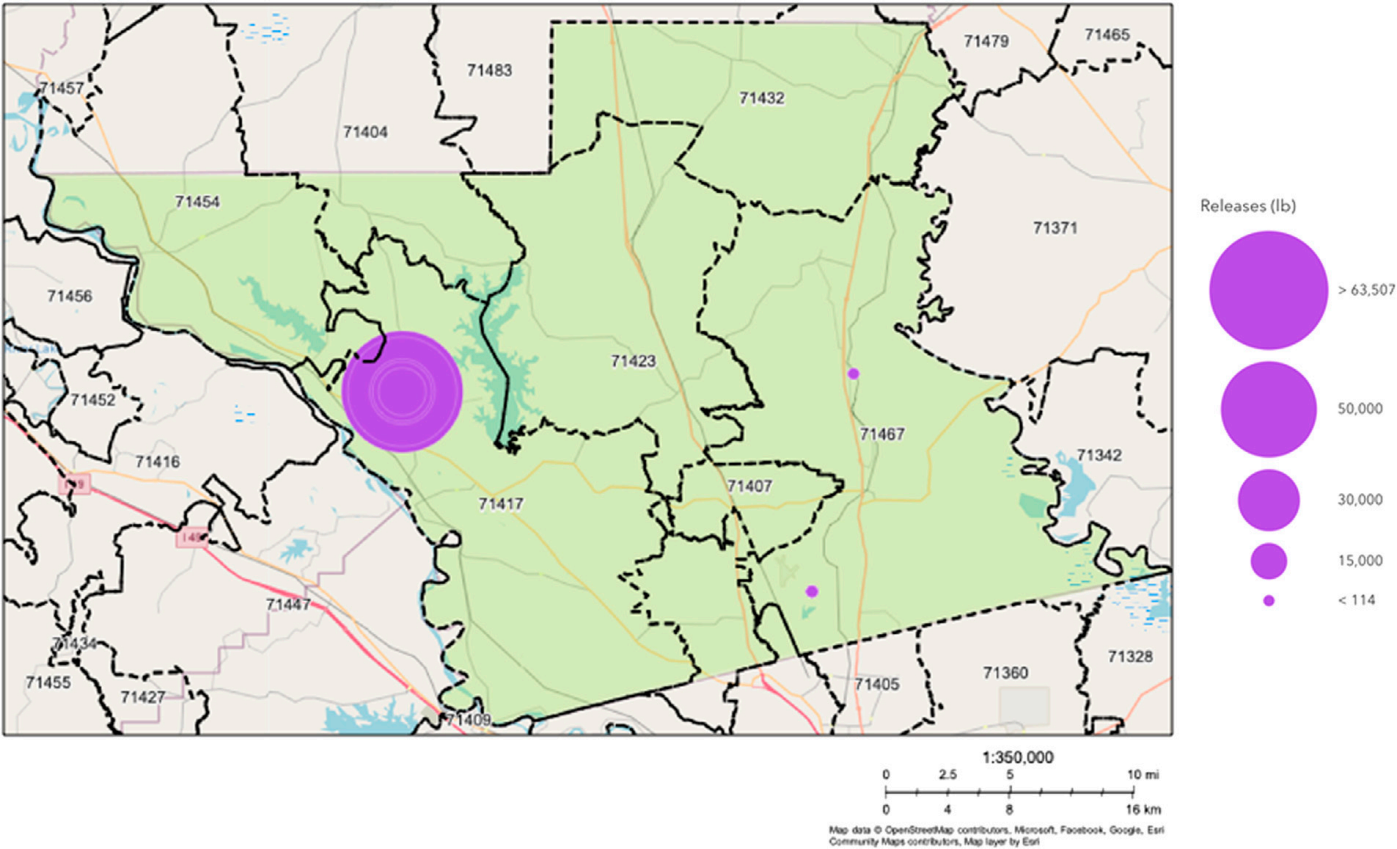
Map from EPA’s Toxic Release Inventory (TRI) showing the location of the thermal-treatment plant at Clean Harbors Colfax, LLC and two other TRI sites in Grant Parish, LA (shown in green). Purple dots show TRI sites with their relative sizes indicating the relative amount of toxic land release in pounds in 2015. Air release is declared to be 0 pounds at the Clean Harbors Site.

A thermal treatment facility operated by Clean Harbors, LLC is located about five miles north of the Colfax town limits (Figure 1). Operational since 1985, it is the only commercially operating OB/OD facility for the thermal treatment of hazardous wastes in the country. Clean Harbors, LLC is one of three sites monitored by the U.S. EPA’s Toxic Release Inventory (TRI) program in Grant Parish, and the only one within the Colfax ZIP code (Figure 1). It produced over 99% of TRI-catalogued pollutants released to the land in the parish (shown for 2015 as an example, but relative proportions are similar for other years) (Table 2). Air releases are declared in the TRI to be zero for Clean Harbors Colfax, indicating that declared releases are landfilled after thermal treatment. Its operations include daily OB/OD of munitions from various sources, which has been noted by the community to cause consistent smoke plumes and noise disturbances [12]. The thermal treatment of explosives is known to generate various toxic byproducts [13], including environmentally persistent free radicals, an emerging class of pollutants associated with particulate matter (PM) that can persist for years in the environment [14, 15]. Three air monitoring samples collected by the Louisiana Department of Environmental Quality (LDEQ) in 2016 also showed that acrolein–a common cardio-, neuro-, nephro-, respiro- and hepato-toxin [16] – levels in the area were above the EPA’s risk-based screening levels [17]. A subsequent cancer risk assessment report by Louisiana Department of Health (LDH) found a higher incidence of colorectal cancer in the census tract that includes Colfax and respiratory cancers in Grant Parish [18].

**TABLE 2:** Toxic Release Inventory (TRI) Land Release data in Colfax (ZIP code 71417) and Grant Parish (2015). Note that Air Release data are listed as 0 in the TRI.

Characteristics	ZIP 71417 (Colfax)	Grant Parish
Number of TRI-reporting facilities within boundaries	1	3
Total chemical release (lb)	63,507.5	64,030.8
Lead and lead compounds (lb)	27,185	27,708
Dioxin and dioxin-like compounds (lb)	0	0.166
Copper and copper compounds (lb)	11,392	11,392
Mercury compounds (lb)	72.5	72.5
Nitroglycerin (lb)	24,858	0

The thermal treatment facility is a focus of community concern, with residents citing various respiratory, cardiovascular, dermal, gastric, thyroid, and other disorders, in addition to cancers [12]. However, no current surveillance data are available at the municipal level that can be used to assess the community’s health concerns. Here, we have used an innovative method aggregating 19 years of hospitalization data to estimate the disease burden in the Colfax ZIP code relative to other areas in Grant Parish and the state using inpatient discharge and mortality records from LDH. The prevalence of respiratory and cardiovascular diseases was estimated based on the inclusion of specific disease codes associated with all-cause hospitalization and mortality. All data were temporally aggregated over 19 years (2000–2018) to preserve patient confidentiality and improve rate stability, considering the small size of the Colfax population. Using these data, we tested the hypothesis that the community of Colfax has a disproportionately high burden of respiratory and cardiovascular diseases compared to the surrounding areas, based on the current literature on the health hazards of exposure to residual hazardous constituents from thermal treatment of explosive and other hazardous wastes.

## Materials and methods

### Population data

Decennial census data for 2000 and 2010 were obtained from the United States Census Bureau (USCB) for the state of Louisiana, Grant Parish, and Grant Parish’s ZIP Code Tabulation Areas (ZCTA; the closest geographic approximation of a ZIP code). As intercensal population estimates are unavailable at the ZCTA-level from USCB for the entire study period, the population for years 2001 through 2018 was extrapolated based on the average annual rate of change in the population for each geography between 2000 and 2010. These individual years of population estimates were then summed for the individual ZCTAs, Grant Parish and the state to create the cumulative population estimate for the 19-year study period.

### Inpatient discharge and mortality records

Based on availability of data when the study was conceived, statewide hospital inpatient discharge and mortality data for the years 2000–2018 were obtained from LDH. Inpatient discharge reporting data often lags by a year; thus, data from 2019 onwards could not be included due to disruptions in the regular reporting system in 2020 due to COVID-19. The 19-year study period was selected to maximize the study size given the small population of Colfax (Table 1). SAS Enterprise Guide^®^ 7.1 was used to extract hospitalization and mortality counts from the respective databases using recorded diagnosis/mortality codes from the International Classification of Diseases (ICD). In the United States, the transition from using ninth-edition (ICD9) to tenth-edition (ICD10) diagnosis codes began in the fourth quarter of 2015; therefore, both ICD9 and ICD10 codes were utilized for this study period. The ICD9 codes used were 390–459 for cardiovascular and circulatory diseases; 460–519 for respiratory diseases; 240–246 for thyroid diseases; and 690–698 for skin inflammation. The ICD10 codes used were I00-I99 for cardiovascular and circulatory diseases; J00-J99 for respiratory diseases; E00-E07 for thyroid diseases; and L20-L30 for skin inflammation. Specific diseases considered included asthma, chronic obstructive pulmonary disease (COPD), lung cancer, respiratory tract infection, hypertension, ischemia, and arrhythmia (Supplementary Material S1). Diseases were selected based on the current evidence of health impact associated with PM exposure as well as community concerns [19, 20]. Estimated prevalence for all disease conditions were calculated from the inpatient discharge data by extracting all records where any of the ICD codes of interest were recorded as a primary or secondary diagnosis code, thus identifying current and underlying respiratory, cardiovascular, thyroid and inflammatory skin diseases among all hospitalized patients. A similar protocol was applied to the mortality data; cases were extracted that recorded the disease of interest (cardiovascular and respiratory) as one of the causes of death. Thyroid and inflammatory skin disorders were excluded from the mortality assessments due to low case counts. Each case was then categorized by the diseases they represented and assigned a geography based on the patient’s ZIP code of residence.

Crude rates of hospitalization and mortality with the diseases of interest were calculated by dividing the number of cases in each geography by the total population and multiplying by a factor of 10,000. These were then adjusted for age, sex assigned at birth (male/female) and race (Black/White) to the 2000 U.S. Standard Population. Age groups used for age-adjustment included 0–11 months, 1–17 years, 18–59 years, 60–74 years and 75 years and older [21]. These age groups were selected based on the differing immunological states of infants, children, young adults and older adults [22, 23]. Other assigned sex and race could not be considered due to very low case counts. Rates were determined for the Colfax ZIP code (71417), Grant Parish excluding the Colfax ZIP code and the state of Louisiana. In order to evaluate differences in cardiovascular and respiratory health outcomes by race and sex, age-adjusted rates were stratified by race (Black and White) and assigned sex (female and male). Certain diseases, including thyroid, skin, asthma and arrhythmia, were excluded from the mortality data analysis due to low case counts. Relative rates were calculated by dividing the Colfax rate by a reference value (i.e., the rate for Grant Parish excluding Colfax or Louisiana). Calculations were conducted using the unconditional maximum likelihood method (‘Wald’) using the *epitools* package (version 5-10.1) in R (4.3.1). All 95% CI for adjusted and relative disease rates were calculated using normal approximation. Differences in disease rates were considered statistically significant if the 95% confidence intervals (CI) of the groups did not overlap). Relative rates where the 95% CI did not include the value of 1.0 was considered statistically significant.

## Results

### Estimated prevalence of cardiovascular, respiratory and other diseases among hospitalized Residents of Colfax, LA

**FIGURE 2:**
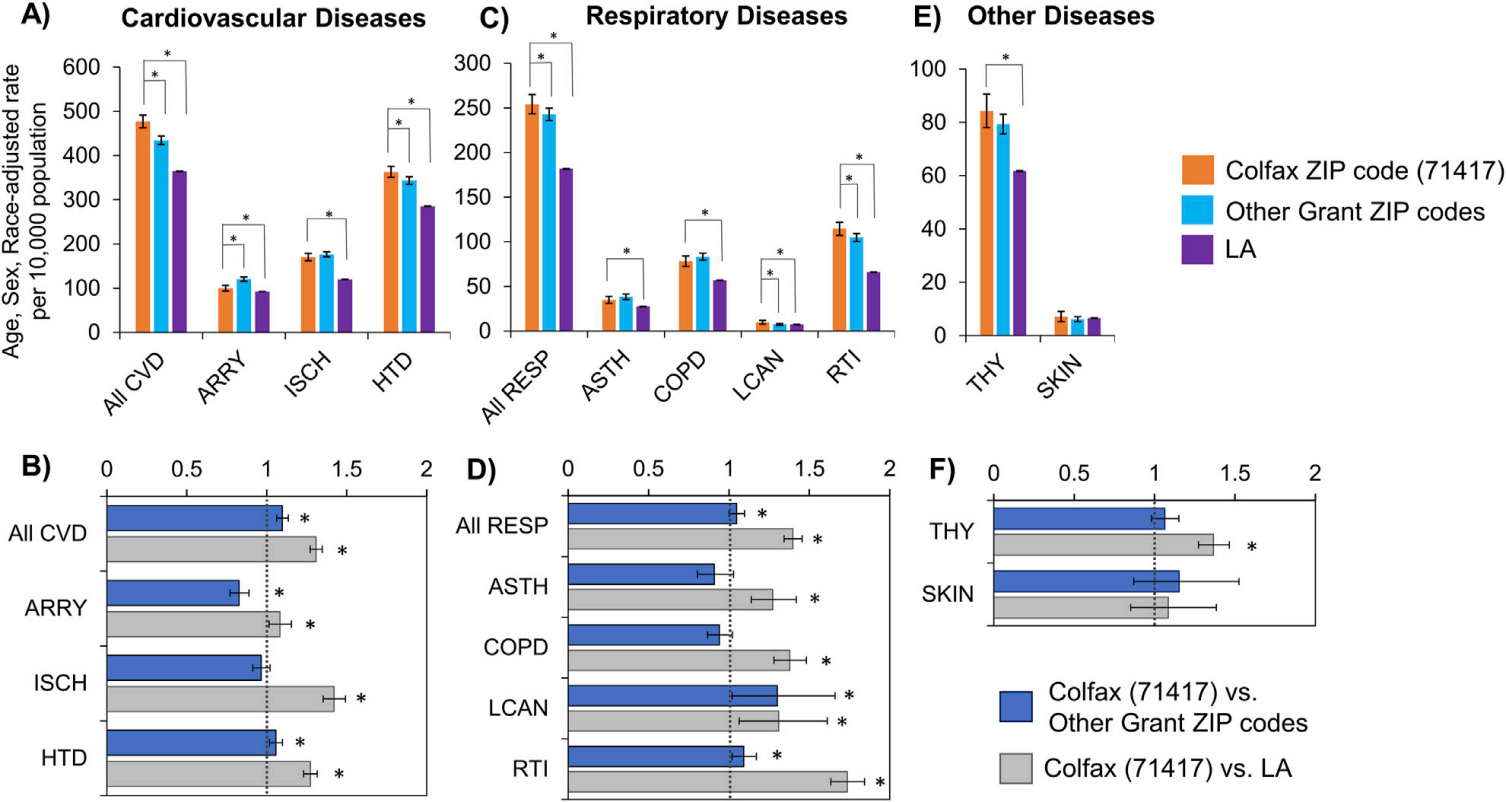
Rates **(A,C,E)** and rate ratios **(B,D,F)** for hospitalizations with cardiovascular **(A,B)**, respiratory **(C,D)**, thyroid and skin **(E,F)** diseases, adjusted for age, sex and race. Statistically significant differences between Colfax and a comparing geography at α = 0.05 are marked with an asterisk (*). CVD, Cardiovascular diseases; ARRY, Arrhythmias; ISCH, Ischemia; HTD, Hypertensive disorders; RESP, Respiratory diseases; ASTH, Asthma; COPD, Chronic Obstructive Pulmonary Disease; LCAN, Lung cancer; RTI, Respiratory Tract Infections; THY, Thyroid diseases; SKIN, Skin diseases.

The rate for presence of cardiovascular disease among hospitalized Colfax residents (2000–2018), adjusted for age, sex and race, was 1.30 times the state average (95% CI: 1.27–1.35; p < 0.0001) and 1.10 times that of Grant Parish excluding Colfax (95% CI: 1.06–1.13; p < 0.0001) (Table 3; Figures 2A,B). Of the specific cardiovascular diseases examined, hypertensive and ischemic disorders followed a similar trend, though the estimated prevalence of ischemia was comparable across all ZIP codes of Grant Parish, and only significantly elevated in Colfax relative to the state average. A contrasting trend was observed for arrhythmia, whose estimated prevalence in Colfax was significantly lower compared to surrounding areas of Grant Parish, though slightly elevated relative to Louisiana.

**TABLE 3:** Estimated prevalence of cardiovascular, respiratory, thyroid and inflammatory skin diseases among hospitalized residents of Colfax (ZIP code 71417), other ZIP codes in Grant Parish, and Louisiana, adjusted for age, sex and race (2000–2018). Age-adjusted rates also presented stratified by race and sex, except for diseases with <=20 cases in Colfax that yielded unstable estimates due to low number of cases.

Health condition	ZIP code 71417 (Colfax)	Other Grant Parish ZIP codes	Louisiana
Age, sex, race-adjusted rates per 10,000 population			
All Cardiovascular^a^ ^,^ ^b^	477	434	364
Arrhythmia^b^ ^,^ ^c^	100	121	92
Ischemia^b^	170	176	120
Hypertension^a^ ^,^ ^b^	363	343	285
All respiratory^a^ ^,^ ^b^	254	243	182
Asthma^b^	35	38	27
COPD^b^	78	83	57
Lung Cancer^a^ ^,^ ^b^	10	7	7
Respiratory Tract Infections^a^ ^,^ ^b^	114	105	66
Thyroid disorders^b^	84	79	62
Skin inflammation	7	6	6
Age-adjusted rates per 10,000 Black residents			
All cardiovascular^a^ ^,^ ^b^	546	341	463
Arrhythmia^a^ ^,^ ^b^	103	87	82
Ischemia^a^ ^,^ ^b^	177	142	121
Hypertension^a^ ^,^ ^b^	467	301	392
All respiratory^a^ ^,^ ^b^	232	200	203
Asthma^a^ ^,^ ^b^	59	46	41
COPD^c^	46	65	52
Respiratory Tract Infections^a^ ^,^ ^b^	106	87	72
Thyroid disorders^a^ ^,^ ^b^	53	31	42
Age-adjusted rates per 10,000 White residents			
All cardiovascular^a^ ^,^ ^b^	473	434	354
Arrhythmia^c^	102	126	96
Ischemia^b^	174	184	123
Hypertension^b^	350	334	272
All respiratory^a^ ^,^ ^b^	262	242	181
Asthma^b^	30	34	25
COPD^b^	85	87	59
Respiratory Tract Infections^a^ ^,^ ^b^	118	107	66
Thyroid disorders^b^	88	81	65
Age-adjusted rates per 10,000 Female residents			
All cardiovascular^a^ ^,^ ^b^	549	465	400
Arrhythmia^b^ ^,^ ^c^	102	124	88
Ischemia^b^	159	152	104
Hypertension^a^ ^,^ ^b^	442	367	321
All respiratory^b^	271	269	202
Asthma^a^ ^,^ ^b^	58	49	42
COPD^b^ ^,^ ^c^	65	94	58
Respiratory Tract Infections^b^	119	116	71
Thyroid disorders^b^ ^,^ ^c^	109	120	84
Age-adjusted rates per 10,000 Male residents			
All cardiovascular^a^ ^,^ ^b^	514	415	426
Arrhythmia	119	128	113
Ischemia^b^	214	219	164
Hypertension^a^ ^,^ ^b^	389	309	330
All respiratory^a^ ^,^ ^b^	262	223	202
Asthma	24	24	21
COPD^b^	88	82	64
Respiratory Tract Infections^a^ ^,^ ^b^	119	101	77
Thyroid disorders^b^	48	42	37

^a^ Colfax rate significantly higher than the rate for other ZIP codes in Grant Parish (P < 0.05).

^b^ Colfax rate significantly higher than the statewide average (P < 0.05).

^c^ Colfax rate significantly lower than the rate for other ZIP codes in Grant Parish (P < 0.05).

The rates for the presence of respiratory disease among hospitalized Colfax residents (2000–2018) were similarly elevated compared to surrounding areas, after adjusting for age, sex and race (Table 3; Figures 2C,D). The Colfax rate for the study period was 1.40 times the state rate (95% CI: 1.34–1.45; p < 0.0001), and approximately 5% higher than the parish rate excluding Colfax (p < 0.047). The four specific respiratory diseases evaluated (asthma, COPD, lung cancer and respiratory tract infections) followed a similar trend where the Colfax rate was 27%–73% higher than the state rate, but similar to the parish rate excluding Colfax.

After adjusting for age, sex and race, rates for thyroid disorders among hospitalized residents of Colfax was slightly but not significantly higher than that of other residents of Grant Parish; however, it was significantly higher than the statewide average by 36% (P < 0.0001). No significant difference was noted regarding skin disorders between residents of Colfax and other areas of the parish or the state (Table 3; Figures 2E,F).

**FIGURE 3:**
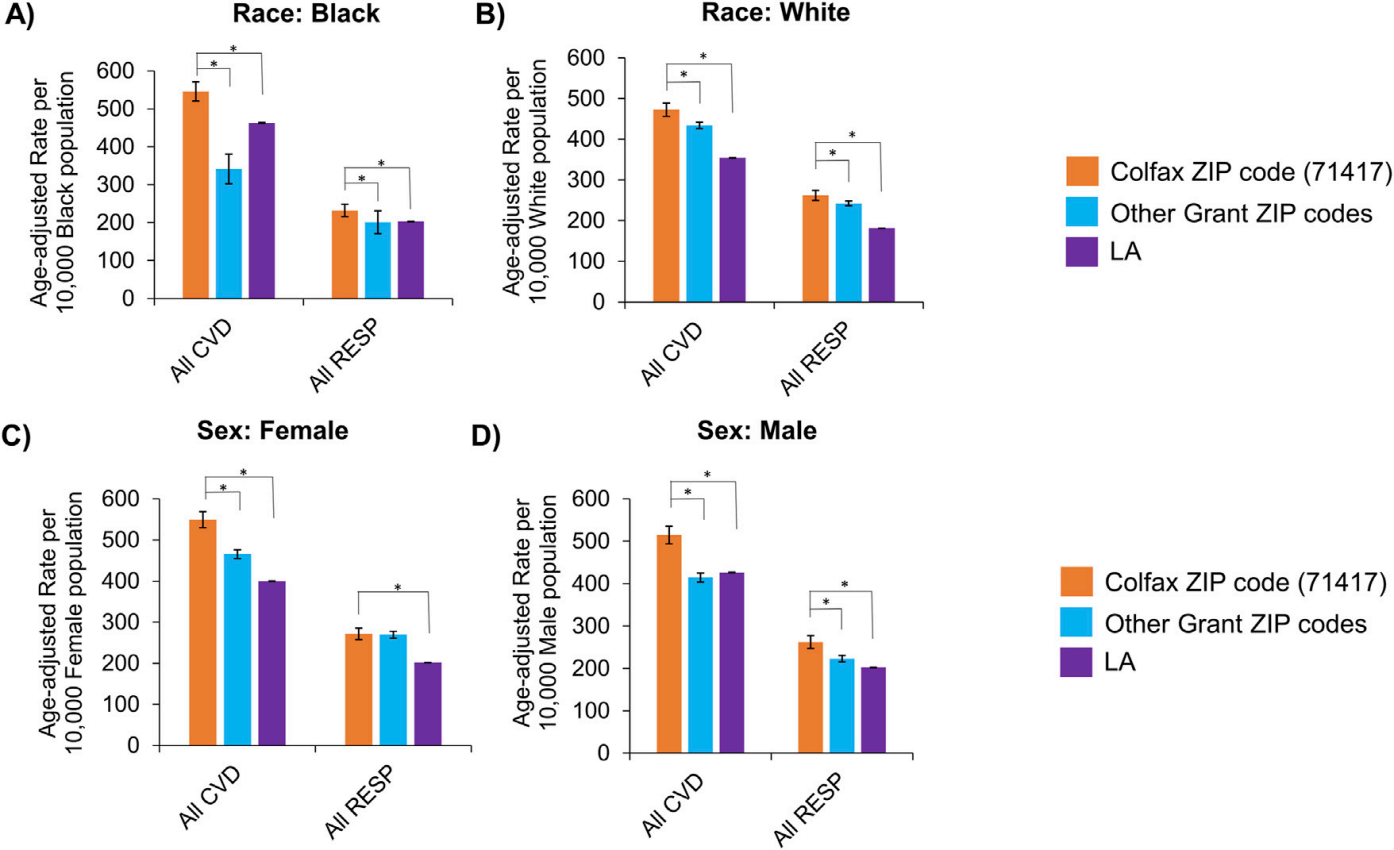
Age-adjusted rates of hospitalizations with any cardiovascular (CVD) or respiratory (RESP) disease, stratified by race **(A,B)** and assigned sex **(C,D)**. Race categories shown include Black **(A)** and White **(B)**; other groups could not be examined due to small sample sizes. Sexes apart from female **(C)** and male **(D)** that may have been assigned also could not be assessed for the same reason. Statistically significant differences between Colfax and a comparing geography at α = 0.05 are marked with an asterisk (*). Error bars represent the 95% confidence interval.

Age-adjusted rates for all diseases were also stratified by race and sex to identify disparities among subgroups (Table 3). The burden of cardiovascular and respiratory diseases overall remained higher in Colfax regardless of race or sex (Figure 3). However, when individual disease rates were examined by race, Black residents of Colfax exhibited a higher burden of disease relative to Black population elsewhere in Grant Parish whereas rates were often comparable for White persons across all ZIP codes in Grant Parish (Supplementary Figure S1). Some differences were noted by sex, as well. Differences in overall respiratory disease prevalence between Colfax and other areas of Grant Parish was noted among males but not females (Figure 3). However, a difference in asthma prevalence between Colfax and other areas of Grant Parish was noted among females and not males (Supplementary Figure S1).

### Estimated mortality resulting from cardiovascular and respiratory diseases among Residents of Colfax, LA

**FIGURE 4:**
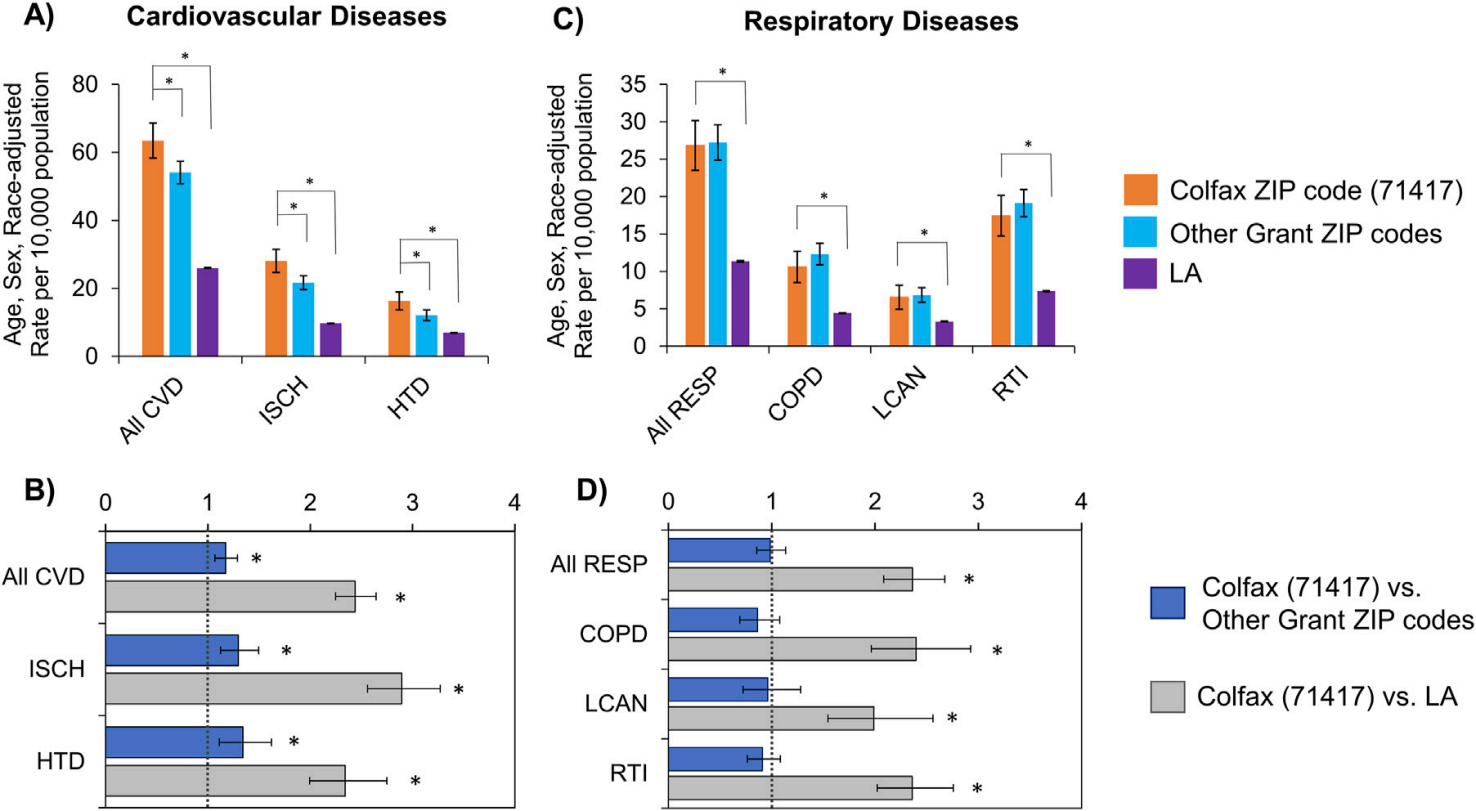
Rates **(A,C)** and rate ratios **(B,D)** for mortality, where a cardiovascular **(A,B)** and respiratory disease **(C,D)** was recorded as a cause of death, adjusted for age, sex and race. Statistically significant differences between Colfax and a comparing geography at α = 0.05 are marked with an asterisk (*). CVD, Cardiovascular diseases; ISCH, Ischemia; HTD, Hypertensive disorders; RESP, Respiratory diseases; COPD, Chronic Obstructive Pulmonary Disease; LCAN, Lung cancer; RTI, Respiratory Tract Infections. Rates for arrhythmia- and asthma-related mortality were unstable due to small counts; hence not shown.

The cardiovascular mortality rate in Colfax (2000–2018), adjusted for age, sex and race, was 2.44 times the state rate (95% CI: 2.24–2.64; p < 0.0001), and 1.17 times the parish rate excluding Colfax (95% CI: 1.07–1.29; p = 0.0009) (Table 4, Figures 4A,B). Of the specific cardiovascular diseases examined, mortality rates with hypertension and ischemic events were significantly higher than those of surrounding regions. Deaths in Colfax, where hypertension was recorded as a cause of death, was 1.34 times that of the rest of Grant Parish (95% CI: 1.11–1.62; p = 0.002) and 2.34 times that of the state (95% CI: 1.99–2.75; p < 0.0001). Similar trends were observed for ischemia as well.

**TABLE 4:** Estimated mortality adjusted for age, sex and race, where cardiovascular and respiratory diseases were recorded as a cause of death, in Colfax (ZIP code 71417), other ZIP codes in Grant Parish and Louisiana (2000–2018). Age-adjusted rates also presented stratified by race and sex. Diseases with ≤20 cases in Colfax, LA excluded as they yielded unstable estimates due low case counts.

Cause of death	ZIP code 71417 (Colfax)	Other Grant Parish ZIP codes	Louisiana
Age, sex, race-adjusted rates per 10,000 residents			
All Cardiovascular^a^ ^,^ ^b^	63	54	26
Ischemia^a^ ^,^ ^b^	28	22	10
Hypertension^a^ ^,^ ^b^	12	16	7
All respiratory^b^	27	27	11
COPD^b^	11	12	4
Lung Cancer^b^	6	7	3
Respiratory Tract Infections^b^	17	19	7
Age-adjusted rates per 10,000 Black residents			
All cardiovascular^a^ ^,^ ^b^	81	49	32
All respiratory^a^ ^,^ ^b^	30	21	11
Age-adjusted rates per 10,000 White residents			
All cardiovascular^a^ ^,^ ^b^	62	55	25
All respiratory^b^	27	28	12
Age-adjusted rates per 10,000 Female residents			
All cardiovascular^a^ ^,^ ^b^	61	51	24
Ischemia^a^ ^,^ ^b^	23	18	8
Hypertension^b^	15	12	7
All respiratory^b^	25	25	10
COPD^b^	9	12	4
Lung Cancer^b^	5	5	2
Respiratory Tract Infections^b^	16	18	6
Age-adjusted rates per 10,000 Male residents			
All cardiovascular^a^ ^,^ ^b^	75	57	31
Ischemia^a^ ^,^ ^b^	37	26	13
Hypertension^a^ ^,^ ^b^	20	12	8
All respiratory^b^	31	30	14
COPD^b^	12	15	5
Lung Cancer^b^	9	10	4
Respiratory Tract Infections^b^	20	23	9

^a^ Colfax rate significantly higher than the rate for other ZIP codes in Grant Parish (P < 0.05)

^b^ Colfax rate significantly higher than the statewide average (P < 0.05)

The mortality rate in Colfax for all respiratory disease conditions was 2.36 times the state rate (95% CI: 2.08–2.68; p < 0.0001) (Table 4, Figures 4C,D). This pattern was consistent for the individual respiratory health conditions examined from the mortality records, including COPD, lung cancer and respiratory tract infections. No major differences were observed for Colfax relative to other Grant Parish ZIP codes.

**FIGURE 5:**
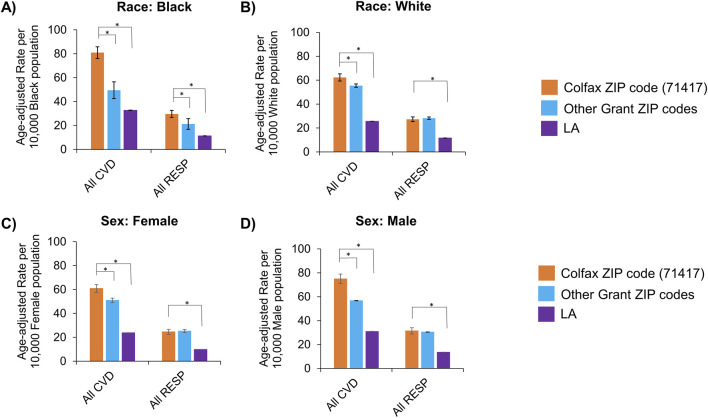
Age-adjusted mortality rate due to any cardiovascular (CVD) or respiratory (RESP) disease, stratified by race **(A,B)** and assigned sex **(C,D)**. Race categories shown include Black **(A)** and White **(B)**; other groups could not be examined due to small sample sizes. Sexes apart from female **(C)** and male **(D)** that may have been assigned also could not be assessed for the same reason. Statistically significant differences between Colfax and a comparing geography at α = 0.05 are marked with an asterisk (*). Error bars represent the 95% confidence interval.

Age-adjusted mortality rates in Colfax were also examined stratified by race, and compared for people of the corresponding race residing in surrounding areas and the state. Among Black Colfax residents (Table 4, Figures 5A,B), the mortality rate with cardiovascular disease was 2.48 times the state rate for Black individuals (95% CI: 2.19–2.80; p < 0.0001), and 1.64 times the rate for Grant Parish excluding Colfax (95% CI: 1.35–1.99; p < 0.0001). Among White individuals (Table 4, Figures 5C,D), the age-adjusted mortality rate with cardiovascular disease was 2.43 times the state rate for White persons (95% CI: 2.20–2.69; p < 0.0001) and 1.22 times the rate for White residents of other areas of Grant Parish (95% CI: 1.003–1.265; p = 0.0443). The respective trends for Black and White individuals were noted for hypertension and ischemia when examined specifically as well.

Age-adjusted mortality rate, with respiratory disease as a cause of death, among Black Colfax residents was 2.63 times the state rate for Black individuals (95% CI: 2.15–3.21; p < 0.0001) and 1.41 times the rate for Grant Parish excluding Colfax (95% CI: 1.04–1.90; p = 0.0269) (Table 4, Figures 5A,B). The mortality rate with respiratory disease for White individuals in Colfax was 2.34 times the state rate for White Louisianans (95% CI: 2.01–2.739; p < 0.0001) but not significantly different from White residents of other parts of Grant Parish. The respective trends by race were consistent when examined for the specific respiratory health conditions: COPD, lung cancer, and respiratory tract infections (Table 4, Supplementary Figure S2).

Similar results were observed when stratified by sex as well. Among females, the age-adjusted mortality rate with cardiovascular disease was 2.56 times the statewide rate for females (95% CI: 2.28–2.87; p < 0.0001) and 1.20 times the rate for other Grant Parish ZIP codes (95% CI: 1.04–1.37; p = 0.0093) (Table 4, Figures 5C,D). The rate ratios for mortality with cardiovascular disease remained comparable in the male population (Table 4, Figures 5C,D). When examined specifically, trends for ischemia were similar to that of cardiovascular diseases overall. However, mortality due to hypertension was only elevated significantly among male residents of Colfax relative to other male population of Grant Parish; no difference was noted for females (Supplementary Figure S2). Significant difference in mortality due to respiratory diseases were noted only between Colfax and Louisiana for both males and females, where the Colfax rate was about twice the state rate for the corresponding population (p < 0.0001). This trend remained consistent for all specific respiratory diseases examined.

## Discussion

In this study, we evaluated the association between residency near a hazardous waste OB/OD facility and hospitalization for cardiovascular, respiratory, thyroid, or skin disease along with mortality from cardiovascular or respiratory disease. This is the first study to examine the impact of living in the 71417 ZIP code (i.e., the community of Colfax, LA) on specific causes of morbidity and mortality. After adjusting for age, sex and race for the study period (2000–2018), our study found a significant increase in cardiovascular and respiratory diseases as a primary or secondary diagnosis at discharge among hospitalized patients in Colfax, LA compared to residents of other ZIP codes of Grant Parish. In addition, residents of Colfax experienced higher mortality with cardiovascular diseases, including ischemia and hypertension. Residents of Colfax were also more likely to have respiratory diseases, like lung cancer and respiratory tract infections, recorded as a diagnosis code, compared to residents of other ZIP codes of Grant Parish. Mortality rates due to respiratory diseases were higher in Colfax than the statewide rate but were similar across Grant Parish (Figures 2, 4).

Since Colfax has a higher proportion of Black residents compared to surrounding areas in Grant Parish, and it is well known that Black Americans tend to face worse cardiovascular and respiratory health outcomes than White Americans [24–26], we compared health outcomes across geographies normalized by race. This analysis revealed disproportionately higher rates of cardiovascular and respiratory diseases overall among both Black and White Colfax residents who were hospitalized, compared to the corresponding population residing elsewhere in the parish (Figure 3). Mortality resulting from cardiovascular diseases was also elevated among Colfax’s Black and White residents compared to residents of the respective race elsewhere in the parish (Figure 5). This indicates that residents of Colfax, regardless of their race, may be affected by environmental factors specific to the area of residence that are not experienced by people living elsewhere in Grant Parish. The data further suggested that Black residents of Colfax experienced greater prevalence and mortality relative to Black people residing elsewhere in Grant Parish, whereas health outcomes were more similar for all White residents across all ZIP codes of Grant Parish (Supplementary Figures S1, S2). Due to the low Black population in other ZIP codes of Grant Parish except Colfax (ZIP code 71417), further investigation is warranted to evaluate potential interactions in place- and race-based disparities for specific disease states in Grant Parish.

Study limitations are balanced by insights gained from our analyses. Hospitalization rates are presented adjusted for age, sex and race at the individual level in an attempt to isolate place-based differences in health outcomes. It is important to emphasize that children and older adults represent particularly vulnerable subpopulations in the context of environmental exposures. Due to their developing or aging physiological systems, these groups are more susceptible to the adverse health effects of airborne pollutants and contaminated water, such as those associated with open burning of hazardous wastes [27, 28]. Children inhale more air per body weight and have immature detoxification systems, while older adults often have pre-existing health conditions that can be exacerbated by environmental stressors [29]. Future studies should prioritize evaluating health outcomes in these age groups to better understand and mitigate the long-term impacts of environmental exposures in rural communities like Colfax. Age-adjusted rates are also stratified by race and sex to show differences across geographies within each sub-population. However, we note that hospital discharge data likely underreports the true disease burden, as it only cites data from hospitals within the state and cannot account for either individuals who were not hospitalized at any point during the 19-year study period or those who sought care out-of-state. Our method of estimating disease burden relied on hospital staff recording not just primary but also secondary diagnosis codes for the disease conditions evaluated. Hospital reporting to the state varies annually, potentially causing the database to miss some inpatient encounters. Though many of these limitations (e.g., underreporting and missing data) could result in an underestimation of morbidity and mortality, it is expected that most sources of error would be consistent across the state and therefore the relative difference between geographies would remain unaffected. Even if underreporting were more severe in rural areas, our detection of differences between the Colfax ZIP code and the state would only be an underestimation. In other words, the differences between Colfax and the state of Louisiana would be even larger if urban-rural effects on data quality were removed. This speaks to the strength of our approach to geographic comparison involving rural areas in the absence of having a sufficiently large sample population for estimating exposure-response relationships.

High spatial resolution is essential in environmental health disparities studies to effectively identify correlations between pollution, health outcomes, and demographic characteristics. [30]. The rural nature of Colfax required use of the entire 71417 ZIP code to protect the identity of individuals included in the hospital database. The 71417 ZIP code is 365 km^2^ in area, and the OBOD facility is 25 km from the southeast corner of the ZIP code. Moreover, the meteorological characteristics of the area are often stable, with high humidity and calm wind conditions 48% of the time over the study duration. Winds flow from the south 24% of the time, and winds emanate from all other directions with roughly equal frequency over the remaining 28% of the time [31]. As a retrospective community health study, we did not collect air pollution surface measurements, as attributing spatially resolved exposures to health outcomes among the 71,417 residents would require detailed information on individuals’ residential history, occupational exposures, and other relevant factors. Although lack of environmental data and coarse resolution are limitations, comparison with the remainder of Grant Parish and with the remainder of the State of Louisiana allowed for some exploration of the role of source proximity in exposures among different population subgroups. This more coarse resolution was still sufficient to detect a statistically significant effect.

Other study limitations involve the lack of available data surrounding genetic or lifestyle factors (e.g., smoking, exercise), most social determinants of health (e.g., poverty status, housing and food insecurity, and preventative healthcare access), indoor/outdoor environmental exposures arising from industrial or agricultural activities, and length of time lived in the specified area of residence at the time of hospitalization—all of which can affect hospitalization and mortality rates, especially in rural areas [32]. Combined with the retrospective ecological design and lack of contemporaneous local air pollutant data or exposure assessments in the area, no correlation or causal inference studies could be performed between the disease burden and known anthropogenic sources of fine PM pollution in the region (e.g., biomass burning for land management [33]). Despite the limitations, it is apparent that Colfax, LA suffers a disproportionate disease burden compared to the rest of Grant Parish and the state. We speculate that exposure to hazardous air pollutants contributes to the disease burden in Colfax, which is consistent with the previously observed increased cancer rates [18] and acrolein levels reported by LDH [34]. Future studies analyzing long-term longitudinal pollutants present in the area and further investigation into potential underlying causes of the increased cardiovascular and respiratory disease burden would greatly benefit the community.

Further, we emphasize the need to develop rigorous methods for analyzing the effects of localized pollution sources for similarly small and potentially at-risk locations like Colfax, LA. Future studies need to focus on geocoding street addresses to a particular point location, possibly combined with population survey and interview data (i.e., mixed methods approaches), to be able to conduct epidemiological studies at smaller scales in rural places, while protecting patient privacy. Use of newer technology may help to identify places that are comparable to Colfax in terms of socioeconomic and demographic characteristics but dissimilar in terms of its environmental characteristics, which could then help to isolate the attributable fraction of the environment on the observed disease burden. Additional resources should be devoted to designing and implementing prospective cohort studies in the area, in collaboration with the community members. By carefully documenting individual health metrics, exposures, potential confounding factors and tracking health outcomes longitudinally, it is more likely that causal inference can be drawn. In the meantime, greater efforts need to be made to ensure at-risk community members have the resources to manage their underlying health conditions. Finally, use of artificial intelligence and machine learning may help public health and research professionals test possible interventions as simulations conducted on synthetic populations that then help to identify specific interventions that can improve health outcomes in the area.

## Conclusion

By calculating the estimated prevalence from the inpatient discharge database, we report a higher burden of cardiovascular and respiratory diseases in the rural community of Colfax, Louisiana (2000–2018), after adjusting for age, sex and race. Mortality associated with these conditions was also significantly higher in Colfax compared to other areas of Grant Parish and Louisiana. These data demonstrate the need for more in-depth studies to understand the differences in rates of hospitalizations and mortality associated with living in the Colfax ZIP code (71417) and to determine if these health impacts are associated with air pollution emitted from the nearby open burning of hazardous wastes. This study also highlights the importance of developing novel methods to estimate disease prevalence in rural regions where low population density leads to data exclusion due to privacy concerns, low statistical power, and data instability, thereby hindering precise prevalence measurements. Further, the lack of infrastructure for consistent, long-term environment and health measurements pose additional challenges specific to addressing environmental health disparities in small, rural populations. Overcoming these challenges will require innovative statistical solutions that help to make “no community…invisible” [35].

## Data Availability

The data analyzed in this study is subject to the following licenses/restrictions: The raw data underlying this study are available from the Louisiana Department of Health (LDH), but restrictions apply due to the need to protect patient confidentiality, and as such, the data are not publicly accessible. Aggregated and deidentified data, with appropriate suppression to safeguard privacy, may be obtained from the authors upon reasonable request and with prior approval from LDH. Requests to access these datasets should be directed to https://ldh.govqa.us/WEBAPP/_rs/(S(b4niape4vnpgud4fb4byeyxi))/SupportHome.aspx?sSessionID&equals;&lp&equals;2.

## Supplementary material

SUPPLEMENTARY FIGURE 1 Heat map showing rate ratios for age-adjusted hospitalizations, stratified by race (Black, White) and sex (female, male). Values greater than one (orange) and less than one (green) indicate significantly higher and lower rate in Colfax, respectively, relative to the reference geography, for the sub-population as noted (P < 0.05). Grey shading indicates no statistically significant difference. Asterisks (*) indicate rate ratios based on unstable rates calculated with counts <=20. CVD, Cardiovascular diseases; ARRY, Arrhythmias; ISCH, Ischemia; HTD, Hypertensive disorders; RESP, Respiratory diseases; ASTH, Asthma; COPD, Chronic Obstructive Pulmonary Disease; LCAN, Lung cancer; RTI, Respiratory Tract Infections; THY, Thyroid diseases; SKIN, Skin diseases.

SUPPLEMENTARY FIGURE 2 Heat map showing rate ratios for age-adjusted mortality, stratified by race (Black, White) and sex (female, male). Values greater than one (orange) and less than one (green) indicate significantly higher and lower rate in Colfax, respectively, relative to the reference geography, for the sub-population as noted (P < 0.05). Grey shading indicates no statistically significant difference. Asterisks (*) indicate rate ratios based on unstable rates calculated with counts <=20. CVD, Cardiovascular diseases; ARRY, Arrhythmias; ISCH, Ischemia; HTD, Hypertensive disorders; RESP, Respiratory diseases; ASTH, Asthma; COPD, Chronic Obstructive Pulmonary Disease; LCAN, Lung cancer; RTI, Respiratory Tract Infections; THY, Thyroid diseases; SKIN, Skin diseases.
