# Supplementary figures and images for: A case study of long-term disease burden in a rural community near an open burn facility
Source: Exp Biol Med (Maywood). 2025 Sep 18;250:10710. doi: 10.3389/ebm.2025.10710 (PMC12489822; doi:10.3389/ebm.2025.10710)

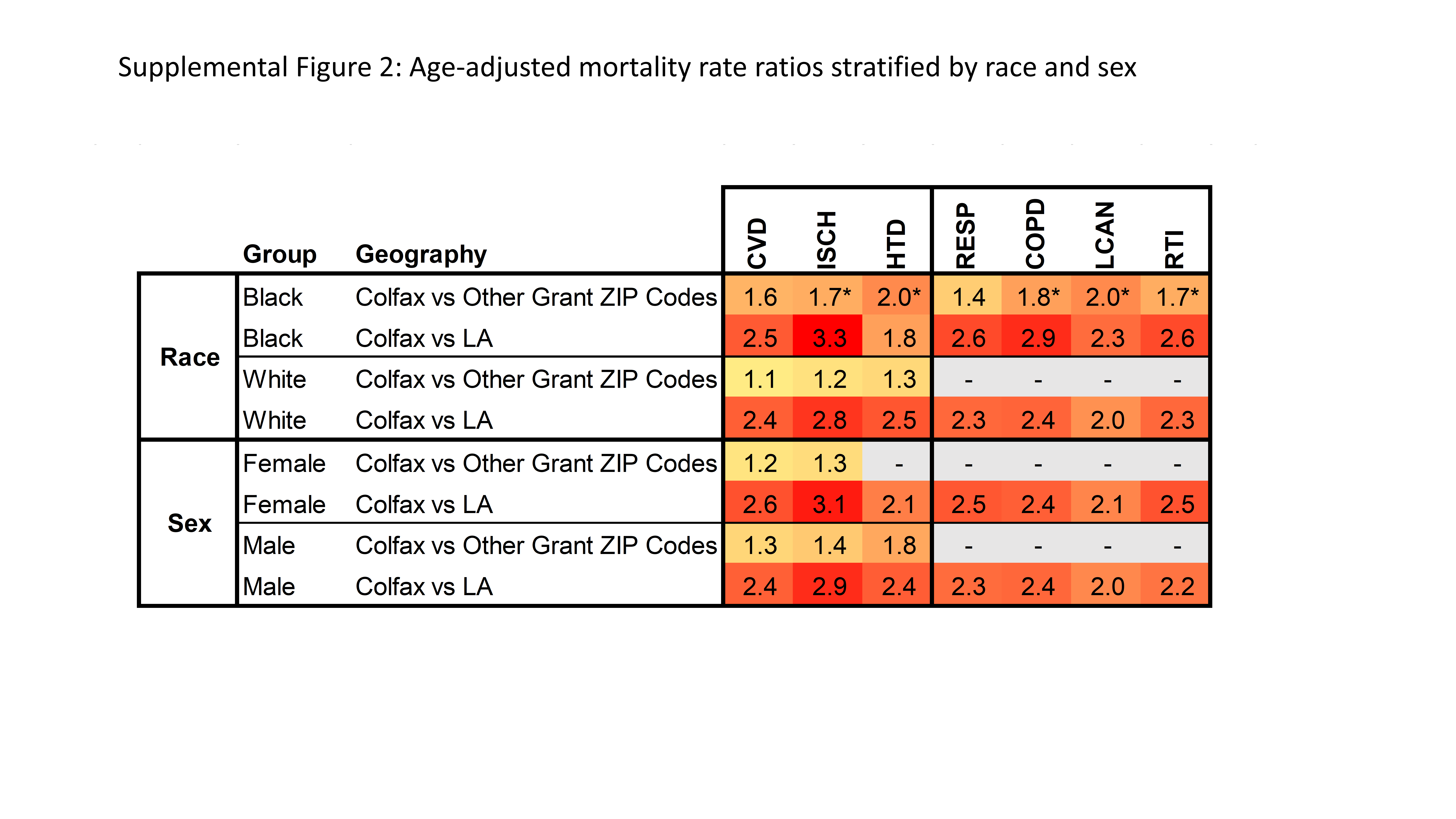

Supplement: Supplementary file 1 [file Image2.tif]

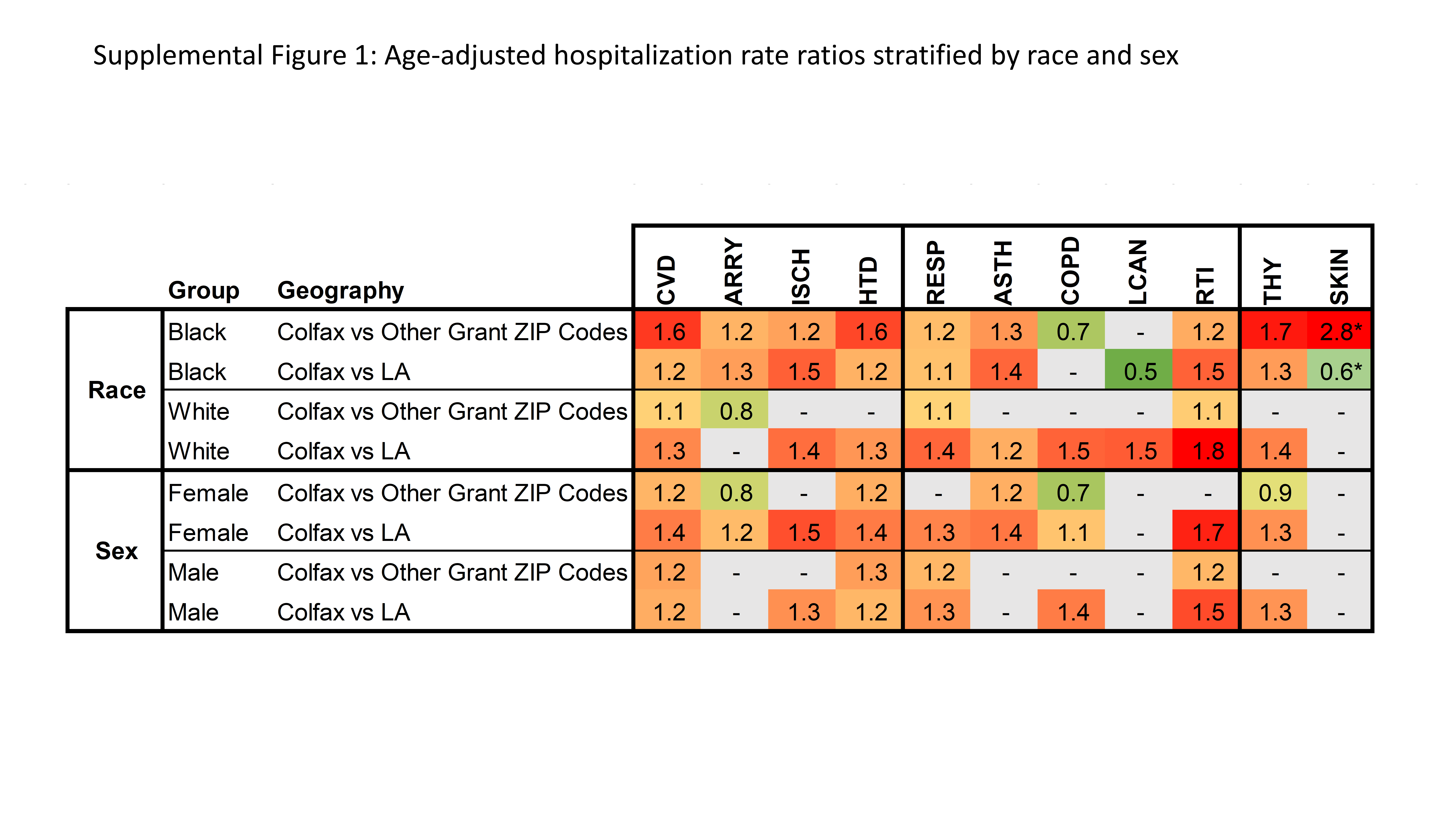

Supplement: Supplementary file 2 [file Image1.tif]
